# Inflammation-associated alterations to the intestinal microbiota reduce colonization resistance against non-typhoidal *Salmonella* during concurrent malaria parasite infection

**DOI:** 10.1038/srep14603

**Published:** 2015-10-05

**Authors:** Jason P. Mooney, Kristen L. Lokken, Mariana X. Byndloss, Michael D. George, Eric M. Velazquez, Franziska Faber, Brian P. Butler, Gregory T. Walker, Mohamed M. Ali, Rashaun Potts, Caitlin Tiffany, Brian M. M. Ahmer, Shirley Luckhart, Renée M. Tsolis

**Affiliations:** 1 Department of Medical Microbiology & Immunology, University of California at Davis, One Shields Avenue, Davis CA 95616; 2 School of Veterinary Medicine, St. George’s University, Grenada, West Indies; 3 Department of Microbiology, The Ohio State University, Columbus, OH 43210 USA; 4 Center for Microbial Interface Biology, The Ohio State University, Columbus, OH 43210 USA; 5 Department of Medical Microbiology and Immunology, Faculty of Medicine, Mansoura University, Mansoura, Egypt; 6 Department of Microbial Infection and Immunity, The Ohio State University, 460 W. 12th Ave, Columbus, OH 43210.

## Abstract

Childhood malaria is a risk factor for disseminated infections with non-typhoidal *Salmonella* (NTS) in sub-Saharan Africa. While hemolytic anemia and an altered cytokine environment have been implicated in increased susceptibility to NTS, it is not known whether malaria affects resistance to intestinal colonization with NTS. To address this question, we utilized a murine model of co-infection. Infection of mice with *Plasmodium yoelii* elicited infiltration of inflammatory macrophages and T cells into the intestinal mucosa and increased expression of inflammatory cytokines. These mucosal responses were also observed in germ-free mice, showing that they are independent of the resident microbiota. Remarkably, *P. yoelii* infection reduced colonization resistance of mice against *S. enterica* serotype Typhimurium. Further, 16S rRNA sequence analysis of the intestinal microbiota revealed marked changes in the community structure. Shifts in the microbiota increased susceptibility to intestinal colonization by *S.* Typhimurium, as demonstrated by microbiota reconstitution of germ-free mice. These results show that *P. yoelii* infection, via alterations to the microbial community in the intestine, decreases resistance to intestinal colonization with NTS. Further they raise the possibility that decreased colonization resistance may synergize with effects of malaria on systemic immunity to increase susceptibility to disseminated NTS infections.

Non-typhoidal serotypes of *Salmonella enterica* (NTS) generally cause a self-limiting diarrheal disease in immunocompetent individuals, however some individuals with immnocompromising conditions are at elevated risk of developing disseminated infection^1^. In sub-Saharan Africa, *S. enterica* serotype Typhimurium and *S. enterica* serotype Enteritidis, are currently among the most common *Salmonella* blood isolates from children^2^,^3^, and treatment of these invasive infections is complicated by the high prevalence of multidrug resistance^4^,^5^,^6^,^7^. A major childhood risk factor in African children for developing systemic NTS infection is malaria^2^,^3^,^8^,^9^. Recent studies have identified multiple factors that may underlie this association, including hemolysis-induced defects in neutrophil function^10^, immunosuppressive effects of malaria on macrophage function^11^, and blunting of inflammatory responses to tissue invasion by NTS^11^,^12^,^13^.

While all of these effects of malaria on the immune response to NTS are observed subsequent to bacterial tissue invasion, it is not known whether malaria affects host resistance to intestinal colonization with NTS upon bacterial ingestion. While not generally considered to be an intestinal infection, malaria is known to affect intestinal function. For example, altered permeability of the intestinal epithelium was observed both in malaria patients and in a murine infection model^14^,^15^. Further, nutrient malabsorption has been reported in severe falciparum malaria^16^, suggesting that the environment within the intestinal lumen could be altered. It has been hypothesized that effects of malaria on the intestine could be secondary to parasite sequestration in the tissue microvasculature, as sequestration has been observed throughout the gastrointestinal tract in malaria patients^17^,^18^,^19^. These findings raised the question, whether effects of malaria on the intestinal microenvironment could affect colonization resistance of the host to NTS. To test this idea, we used a murine model of concurrent malaria and NTS infection to examine the effect of infection with the rodent malaria parasite *P. yoelii nigeriensis* (*P. yoelii*) on the initial phase of intestinal colonization by *S. enterica* serotype Typhimurium (*S.* Typhimurium).

## Results

### *P. yoelii* infection leads to parasite sequestration in the gut microvasculature, epithelial damage and infiltration of mononuclear cells into the lamina propria

**Figure 1:**
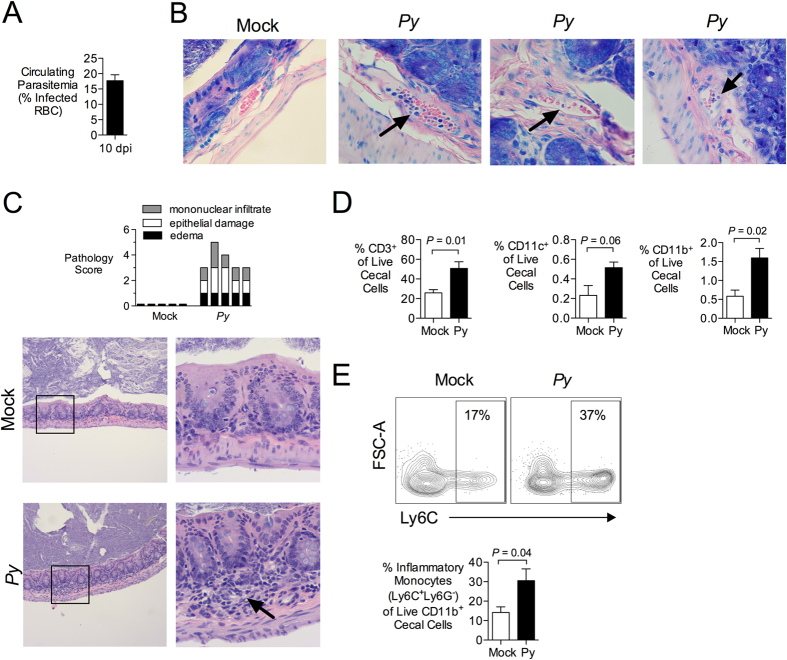
Infection with *Plasmodium yoelii* induces intestinal inflammation. (**A**) C57BL/6J mice were infected intraperitoneally (ip) with 4 × 10^7^ infected red blood cells (iRBC). Parasitemia at 10 days post malaria was determined on Giemsa-stained blood smears and the percentage of iRBC is displayed as mean + SEM for all mice used in panels B–E (n = 10). (**B**) At 10 days post *P. yoelii (Py)* inoculation, iRBC were present in blood vessels of paraffin-embedded cecal tissues stained with Giemsa (n = 3 separate pyn mice). Images were acquired with a 60× objective. Black arrows indicate parasites inside blood vessels. (**C**) Blinded histopathological analysis of *P. yoelii*-induced alterations to the intestinal mucosa. Criteria for scoring are provided in Table S1. Each bar represents an individual mouse (n = 5). Images were acquired with a 10× objective (left panel) and 40× (right panel). Arrow indicates mononuclear inflammation. (**D**,**E**) Flow cytometric analysis of cell suspensions from cecal mucosa obtained 10 d after *P. yoelii* inoculation. (**D**) Percentage of CD3, CD11b or CD11c expressing live, singlet cecal cells. Mean + SEM (n = 5). (**E**) Percent of Inflammatory Monocytes (Ly6G− Ly6C+) within the fraction of live, singlet cecal cells expressing CD11b. Mean + SEM (n = 5). Gating strategy and additional representative dot plots are shown in Fig. S1. Significance for differences between experimental groups was determined using Student’s *t* test on logarithmically transformed data. Mice were housed in groups of 4–5 per cage.

To study effects of acute malaria parasite infection on the intestine, C57BL/6 mice were inoculated with *P. yoelii*. Mice developed parasitemia, measured as the percentage of red blood cells harboring parasite, that peaked at maximal levels between days 10 and 15 after inoculation. We selected this phase of maximal parasitemia to interrogate effects of malaria on the intestine (Fig. 1A). During this time, *P. yoelii*-infected blood cells could be observed in the intestinal microvasculature, with evidence of sequestration on the vascular endothelium (Fig. 1B). Blinded histopathology analysis of the large intestinal wall at the cecum revealed mild but significant changes, including edema of the lamina propria, focal loss of goblet cells, hyperplasia of undifferentiated enterocytes, and focal infiltration of mononuclear cells into the lamina propria, but no evidence of inflammatory cell exudation into the intestinal lumen (Fig. 1C).

Analysis of the cellular infiltrates by flow cytometry revealed that they consisted primarily of T cells (CD3+), as well as CD11b+ and CD11c+ myeloid cells (Fig. 1D and Fig. S1). Approximately 30% of the infiltrating CD11b+ cells exhibited an inflammatory phenotype, as evidenced by expression of Ly6C (Fig. 1E). Together, these results show that acute malaria parasite infection is associated with inflammatory changes in the wall of the intestine.

### Inflammatory changes result from parasite infection, and do not require the endogenous microbiota

**Figure 2:**
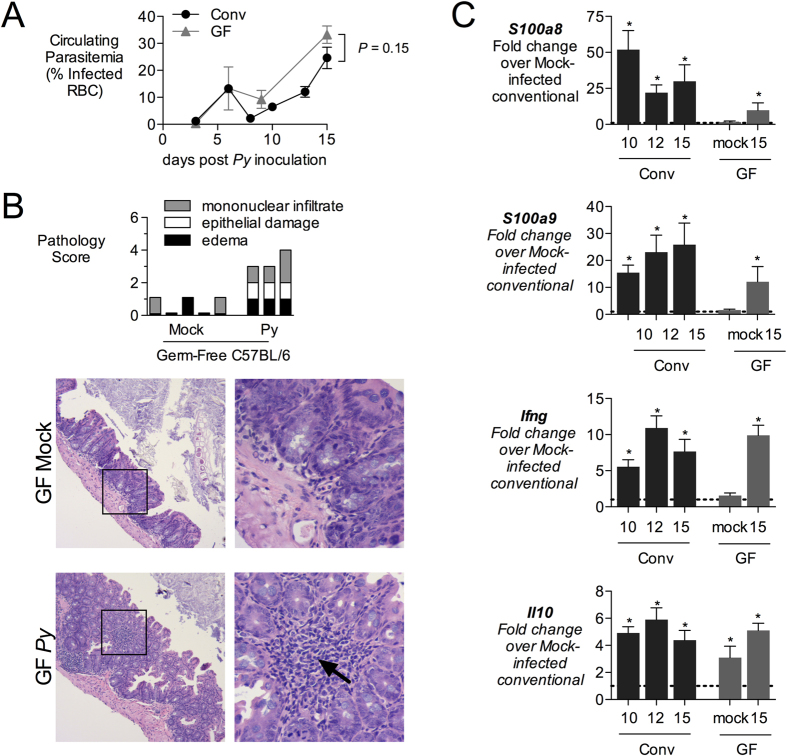
Induction of inflammatory mediators in intestinal mucosa. (**A**) conventional (Conv) or germ-free (GF) C57BL/6J mice were inoculated intraperitoneally (ip) with blood containing 4 × 10^7^ infected red blood cells (iRBC). Parasitemia was determined on Giemsa-stained blood smears and the percentage of iRBC is displayed as mean + SEM (Conv, n = 5 − 11; GF, n = 3). (**B**) Blinded histopathological analysis was performed on cecal tissue obtained from GF mice at 15d post inoculation, as outlined in Table S1. Each bar represents an individual mouse. Images were acquired with 10× (left panels) and 40× objectives (right panels). Arrow indicates mononuclear infiltration. (**C**) Expression analysis of inflammatory markers by qRT-PCR. Transcript levels of calprotectin (subunits *S100a8* and *S100a9*), interferon gamma (*Ifng*) and interleukin-10 (*Il10*) were determined in cecal tissue from Conv or GF mice sacrificed at 10, 12 or 15 d after *P. yoelii* inoculation. Data shown as fold-change over mock-treated Conv mice (indicated with dashed line at 1) with mean + SEM for (Conv, n = 5 − 11; GF, n = 3). Asterisk (*) indicates significance (P < 0.05) when compared to mock-treated mice as determined by Student’s *t* test on logarithmically transformed data, (ns) indicates no significance (P > 0.05). Groups of mice were co-housed.

We next interrogated whether the mucosal inflammation observed in the *P. yoelii*-infected mice resulted from the parasite infection itself, or rather from resulting penetration of the endogenous intestinal microbiota into the tissue. To address this question, we compared intestinal responses of conventionally reared or germ-free C57BL/6 mice during maximal parasitemia with *P. yoelii* (Fig. 2A). Both groups of mice exhibited similar levels of parasitemia at d15 after inoculation, so this time point was used for comparison (Fig. 2A). Histopathology scoring revealed a comparable severity of histologic changes in *P. yoelii*-infected germ-free mice compared to conventional mice (Fig. 2B and Fig. 1C). Based on our observation (Fig. 1) that inflammatory infiltrates in the intestine of conventional mice contained T cells and macrophages, we analyzed mucosal expression of *S100a8* and *S100a9*, produced by inflammatory macrophages during malaria^20^, as well of IL-10 and interferon gamma, produced by T cells^21^ (Fig. 2B). In conventional mice, expression of *S100a8*, *S100a9*, *Il10* and *Ifng* was elevated at d10 and d15 after *P. yoelii* infection. A similar pattern of induction was observed in germ-free mice evaluated at d15. There was a nonsignificant trend (*P* > 0.05) for lower induction of *S100a8* and *S100a9* in the germ-free mice. These results suggest that while an intact intestinal microbiota may contribute to inflammatory changes in the gut mucosa during *P. yoelii* infection, it is not required for this effect, rather it is the malaria parasite infection driving this inflammatory response.

### The composition of the intestinal microbiota is altered during malaria parasite infection

**Figure 3:**
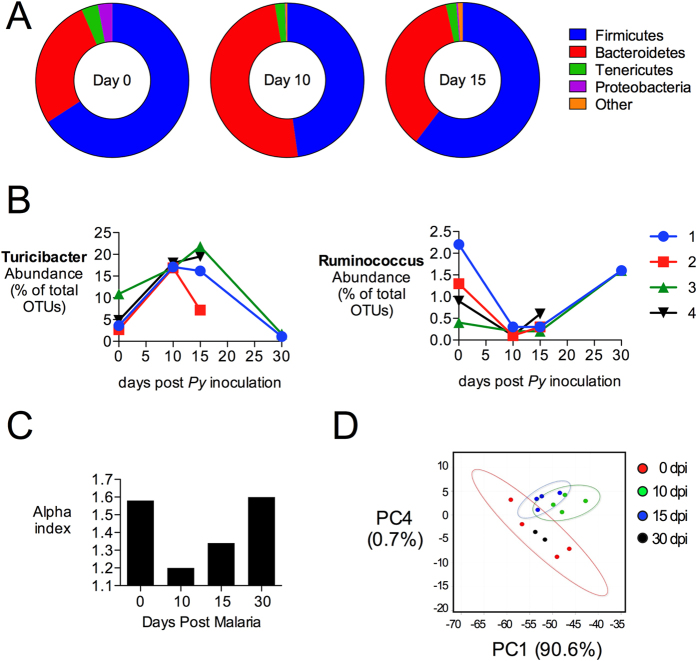
Changes in microbial communities after *P. yoelii* infection. Illumina MiSeq analysis of 16S rRNA amplicons in fecal DNA extracts from *P. yoelii*-infected mice. (**A**) Average abundance of microbial communities at the phylum level as determined by percent OTU readings (n = 4) at days 0, 10 and 15. OTU with significant changes are shown in Table 1. Results for individual animals and mock-treated controls are shown in Fig. S2. (**B**) Abundance of *Turicibacter* and *Ruminococcus* (genus) before and after parasite inoculation. Each line represents individual mice (1–4) with two mice succumbing to morbidity after day 15. (**C**) Alpha diversity of 16S rRNA sequences at different time points after *P. yoelii* infection, determined using Explicet^46^. (**D**) Principal component analysis at the genus level, plotted using METAGENassist^44^. Groups of mice were co-housed.

To determine whether malaria parasite infection impacts the resident microbiota, fecal pellets were collected from two groups of co-housed C57BL/6 mice before inoculation with *P. yoelii* and at days 10, 15 and 30 days post infection. Illumina MiSeq analysis of amplicons from the 16S rRNA locus in fecal DNA extracts revealed significant alterations in the colonic microbiota (Fig. 3). At the phylum level, a decreased abundance of Firmicutes and a relative increase in the abundance of Bacteroidetes were observed at d10 (Fig. 3A). These changes were not simply the result of fluctuation in the resident microbiota over time or of husbandry-related effects, since a group of mock-infected mice from the same colony exhibited a stable microbiota composition over time (Fig. S2). At the genus level, acute malaria parasite infection at d10 was associated with an increase in the relative abundance of unclassified members of the Rikenellaceae (*P* = 0.009), Ruminococcaceae (*P* = 0.007), and Bacteroidales (*P* = 0.024), as well as of *Turicibacter* (*P* = 0.001). A decrease in *Ruminococcus* was also noted (*P* = 0.041; Fig. 3B and Table 1). Since the *P. yoelii*-infected mice were co-housed, we cannot formally exclude a contribution of coprophagy to the altered fecal microbiota. However one co-housed mouse in this group (not shown) was not infected with *P. yoelii* and did not exhibit these alterations in the fecal microbiota, suggesting that coprophagy alone is not sufficient to alter the microbiota in a conventionally-reared mouse. Overall, as *P. yoelii* infection progressed, the diversity of the fecal microbiota decreased by d10, with a gradual recovery by d30 after infection (Fig. 3C). At day 30, after resolution of infection, the composition of the microbiota most closely resembled the composition prior to infection, as shown by principal component analysis (Fig. 3D), suggesting that the effect of malaria parasite infection on microbial communities in the large intestine is transient.

### Malaria parasite infection lowers the implantation dose for *S.* Typhimurium in mice

**Figure 4:**
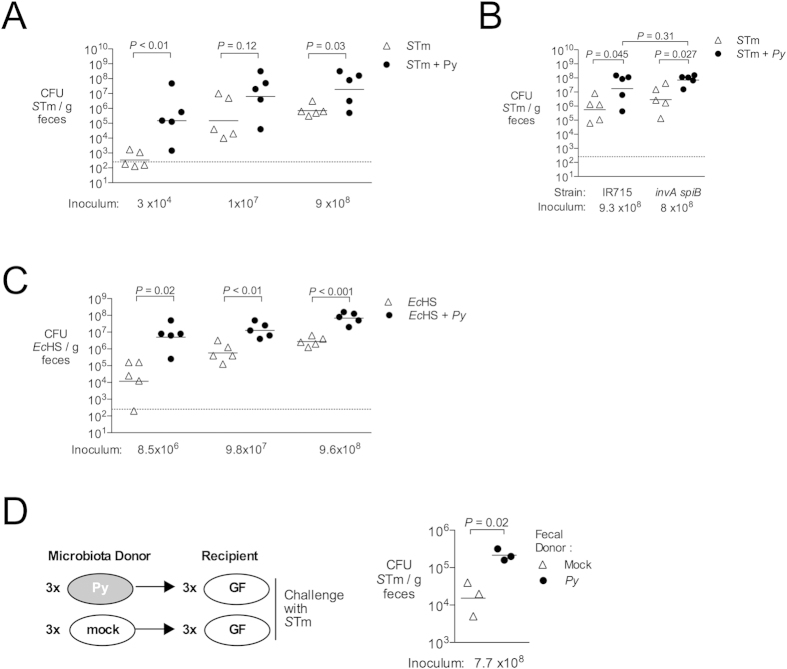
Increased intestinal colonization of *S*. Typhimurium and *E. coli* during malaria parasite infection. (**A**–**C**) To determine effects of malaria on colonization of mice with *E. coli* and *S.* Typhimurium, C57BL/6 mice were inoculated intragastrically with bacteria at 10 days after *P. yoelii* infection or mock treatment. Fecal pellets were collected 24 h post challenge for determination of fecal CFU. Groups of mice were co-housed. (**A**) Effect of *P. yoelii* infection on cecal colonization of *S.* Typhimurium (STm) strain IR715. (**B**) Effect of *P. yoelii* infection on shedding of STm strain IR715 or an isogenic *invAspiB* mutant in feces (**C**) Effect of *P. yoelii* infection on fecal CFU of human commensal *E. coli* strain HS (EcHS). (**D**) Susceptibility of germ-free mice reconstituted with colonic microbiota from *P. yoelii*-infected or control mice to colonization with *S.* Typhimurium. Each reconstituted mouse was housed individually for the duration of the experiment. Each symbol represents an individual mouse, with horizontal bars representing the geometric mean. Dashed lines indicate limit of detection. Significance of differences between experimental groups was determined using a Student’s *t* test on logarithmically transformed data.

The finding that *P. yoelii* infection altered the intestinal microbiota raised the possibility that these changes could affect susceptibility of mice to infection with NTS. To address this question, we determined the dose at which 50% of mice would become infected with *S*. Typhimurium (implantation dose 50 or ID_50_) at 1 day after infection, according to the method of Reed and Muench^22^. In control mice, the ID_50_ for *S.* Typhimurium IR715 was 1.1 × 10^4^ CFU, 34-fold higher than at the peak of *P. yoelii* infection, where this value was reduced to 3.2 × 10^2^ CFU (Table 2 and Fig. S3A). Further, *P. yoelii*-infected mice inoculated with *S.* Typhimurium at varying doses were colonized at significantly higher levels, as assessed by determining CFU in the feces (Fig. 4A). By 4 days after *S.* Typhimurium infection, colonization levels were similar in both groups (data not shown), likely because *S.* Typhimurium infection elicits intestinal inflammation in the control mice, a factor that promotes its outgrowth in the intestinal lumen^23^,^24^,^25^,^26^. However, elevated intestinal colonization of *S.* Typhimurium at 1 day post inoculation in the *P. yoelii*-infected mice was independent of the ability of *S.* Typhimurium to elicit a mucosal inflammatory response, since an *invA spiB* mutant (SPN487), defective in the SPI-1 and SPI-2 encoded type III secretion systems required for mucosal invasion and inflammation^27^,^28^, was also recovered in higher numbers from *P. yoelii*-infected mice (Fig. 4B and Fig. S3B). Further, the human commensal strain *Escherichia coli* HS^29^, which does not cause intestinal inflammation, also colonized the intestine of *P. yoelii*-infected mice at higher levels than in control mice (Fig. 4C), and this elevated colonization was maintained for several days after *E. coli* inoculation (data not shown). Of note, we did not observe an effect of *P. yoelii* infection on colonization of *E. coli* in our 16S microbiota analysis (Fig. 3), most likely because our mice (C57BL/6J) were not consistently colonized with detectable levels of *E. coli*. However, mice inoculated concurrently with *E. coli* and *P. yoelii* exhibited higher colonization of *E. coli* compared to control mice 14 days later (data not shown), implying that if *E. coli* is present at the outset of infection, its outgrowth is promoted during malaria. Together, these results suggest that changes to the intestinal milieu caused by *P. yoelii* infection promote colonization of the intestine with both *S.* Typhimurium and *E. coli*.

### Alterations to the microbiota induced by malaria parasite infection promote colonization with *S.* Typhimurium

To determine the significance of the altered intestinal microbiota for increased *S.* Typhimurium colonization during malaria, we performed a microbiota transfer experiment. Cecal contents were isolated under anaerobic conditions from three control mice and three mice acutely infected with *P. yoelii* at d10 post infection (Fig. S3C), and the contents from each single mouse were transferred to an individual germ-free Swiss-Webster recipient via oral gavage. After allowing 6 days for the microbiota to become established, mice were inoculated via gavage with *S.* Typhimurium. One day later, *S.* Typhimurium colonization was measured via fecal shedding. Figure 4D shows that recipients of the microbiota transplant from *P. yoelii*-infected mice were colonized at a tenfold higher level with *S.* Typhimurium than recipients of the microbiota from control mice. These results suggest that dysbiosis induced by malaria parasite infection lowers colonization resistance of mice against *S.* Typhimurium.

## Discussion

Studies in murine models have shown that multiple responses to malaria parasite infection conspire to increase susceptibility to disseminated infection. Malaria-induced hemolysis impacts maturation of neutrophils, which play a critical role in containing spread of extracellular bacteria^10^. Further, malaria-induced IL-10, that is beneficial in the context of dampening parasite-induced inflammation, has a detrimental effect on control of intracellular *S.* Typhimurium replication within hepatic macrophages^11^. As a consequence, once bacteria have disseminated from the gut, control of systemic infection is compromised. Further, disruption of intestinal barrier function and suppression of NTS-induced neutrophil recruitment to the mucosa may facilitate disseminated infection^12^,^14^. This study identifies a new factor that suppresses resistance to initial colonization of the intestine by *S.* Typhimurium, namely alterations to the community structure of the intestinal microbiota, which outnumbers the body’s own cells by an order of magnitude (Ref 30). As a result, malaria reduces colonization resistance against *S.* Typhimurium–in our model of concurrent infection, the effective dose of bacteria needed to establish intestinal infection was decreased by 97%.

Loss of colonization resistance during malaria did not involve epithelial invasion or induction of inflammation by *S.* Typhimurium, as it was independent of the SPI-1 and SPI-2 Type III secretion systems that are needed for invasion and induction of intestinal inflammation (Fig. 4)^31^. Further, a non-invasive commensal *E. coli* strain also exhibited enhanced colonization in our model, suggesting that perturbation of the microbial community by malaria opens an ecologic niche that can be occupied by either *S.* Typhimurium or *E. coli.* This altered environment was associated with mononuclear infiltration of the intestinal mucosa (Fig. 1 and Fig. 2), suggesting the possibility that inflammatory changes may drive these changes to the microbiota. However, malaria-induced inflammation did not appear to be necessary for loss of colonization resistance, because reduced colonization resistance could be transferred to germ-free mice independently of malaria, by transfer of the cecal microbiota (Fig. 4D). Of note, based on the different types of inflammatory responses observed in the intestinal mucosa, the mechanism by which malaria alters the endogenous microbiota is likely to be different from the mechanism by which *S.* Typhimurium promotes its own outgrowth via inflammation. In our study, we observed an infiltration of T cells and mononuclear phagocytes in *P. yoelii*-infected mice (Fig. 1). In addition, we observed an increase in FcεRI-positive cells, which is consistent with our previous report of an increase in mucosal mast cells in this model (Fig. S1 and^14^). In contrast, in the murine colitis model used to model enteric pathology of *S.* Typhimurium infection, a massive exudation of neutrophils into the mucosa and the intestinal lumen results in production of oxygen and nitrogen radicals that alter the environment and promote outgrowth of *S.* Typhimurium in the gut lumen^25^.

*P. yoelii* infection resulted in a decrease in the complexity of the cecal microbiota, as well as a decrease in the abundance of Firmicutes. Interestingly, members of this phylum have been shown to be decreased after treatment with antibiotics including cefoperazone^32^, shown to reduce colonization resistance against *Clostridium difficile*^32^ and streptomycin, which enhances colonization with *S.* Typhimurium^33^. Further, a decrease in members of the Firmicutes has been observed in patients with inflammatory bowel disease, a condition that is associated with increased colonization of *E. coli*^26^,^34^,^35^,^36^,^37^,^38^. However, whether shared mechanisms underlie outgrowth of *S.* Typhimurium and *E. coli* in each of these conditions is unknown, since the mechanisms linking alterations in the microbiota with reduced colonization resistance are incompletely understood.

Taken together, the results of this study suggest that malaria, via alterations to the intestinal environment, shifts the community structure of the gut microbiota to provide a benefit to colonizing *S.* Typhimurium and *E. coli*.

## Methods

### Plasmodium yoelii nigeriensis (*P. yoelii*)

Parasite stocks were obtained from the Malaria Research and Reference Reagent Resource and the species and strain identities were confirmed by DNA sequencing of merozoite surface protein-1 (MSP-1)^14^. Parasite stocks were prepared by passage in CD-1 mice. For experiments, mice were inoculated intraperitoneally (i.p.) on day 0 with blood containing approximately 4 × 10^7^ infected red blood cells (iRBCs). Mock-treated controls were injected with an equal volume of blood from uninfected CD-1 mice.

### Bacterial strains

*S.* Typhimurium IR715 (pHP45Ω) (JM1), a derivative of ATCC14028 resistant to nalidixic acid (Nal), ampicillin and streptomycin^39^,^40^ or an isogenic *invA spiB* double-mutant (SPN487, ∆*invA*(−9 to +2057) ∆*spiB*(+25 to +1209), Nal^R^), was used for this work^41^. *Escherichia coli* HS (ATCC700891), a human commensal strain resistant to ampicillin, was obtained from ATCC^29^. Bacterial strains used for inoculation of mice were cultured aerobically for 16 h in Lysogeny Broth (LB) with appropriate antibiotic selection.

### Animal experiments

All experiments were performed in accordance with guidelines and regulations as outlined and approved by the UC Davis or Ohio State University Institutional Animal Care and Use Committees (IACUC). *Specific pathogen free (SPF) mice:* 6–8 week-old female C57BL/6J mice were purchased from the Jackson Laboratory (Bar Harbor, Maine) and maintained under SPF conditions. *Germ-free (GF) mice:* GF C57BL/6 and Swiss Webster mice were bred inside germ-free isolators. Experimentation in GF mice was performed in an independent GF isolator and for fecal microbiota reconstitution; mice were transferred to a biosafety cabinet for inoculation and maintained in sterile cages for the duration of the experiment. After reconstitution, GF mice were caged individually.

### Microbial readouts of colonization

Parasite infection was monitored by blood collection from tail snips. Parasitemia was determined by counting the percentage of *Plasmodium yoelii* iRBCs on thin blood smears stained with Giemsa (Acros Organics). For quantification of *S.* Typhimurium or *E. coli*, fecal pellets, collected 1 day after intragastric inoculation, were homogenized and serial dilutions spread on LB agar plates containing appropriate selective antibiotics.

### Histopathology

Histological samples were collected at the time of necropsy. 5 μm sections were cut from formalin fixed paraffin embedded tissues and stained with hematoxylin and eosin or Giemsa by the UC Davis Veterinary Pathology Laboratory. A veterinary pathologist (MXB) performed histopathology scoring in a blinded fashion, according to scoring criteria detailed in Table S1.

### Isolation of Intestinal Cells and Flow Cytometry

Whole ceca were removed aseptically, opened and gently scraped with closed scissors to remove ingesta. Tissue was then stored in 5 mL of RPMI + 5%FBS (Gibco) on ice until processing. Next, tissue was washed 4 times in 20 mL DPBS to remove excess cecal content (Gibco). Ceca were then placed in pre-warmed C-Tubes (Miltenyi) containing 10 mL RPMI + 5%FBS, 30 μL of DNase I (300 units) (Roche) and 21 μL Liberase (0.55 Wunsch units) (Roche). Tubes processed twice on gentleMACS Dissociator (Miltenyi) program Brain 1.01 and incubated, rotating, at 37 °C for 35 min. After enzyme digestion, tubes processed twice on gentleMACS program B.01. Next, cell suspension was filtered through 70 um nylon filter and washed with 10 mL cold DPBS. Cells were finally resuspended in 1 mL DPBS and ready for antibody staining. Cell suspensions were stained with 7 μL of stained with aqua live/dead cell discriminator (no. L34597; Invitrogen) in accordance with the manufacturer’s protocol. Cells were then rinsed and resuspended in 50 μL fluorescence-activated cell sorting (FACS) buffer (PBS containing 1% bovine serum albumin and 1 mM EDTA). Next, cells were blocked with 4 μL anti-CD16/32 antibody (clone 93; eBioscience) for 20 min on ice, covered. Next, cells were stained for 20 min on ice with 4 μg of each of the following antibodies: anti-Ly6C-PacBlue (clone Hk1.4, Biolegend), anti-Ly6G-APC (clone 1A8, Biolegend), anti-CD11b-FITC (clone M1/70, BD biosciences), anti-CD11c-PerCP-Cy5.5 (clone N418, Biolegend), anti-CD3-PE-Cy7 (clone17A2, Biolegend), anti-CD19-AF594 (clone 6D5, Biolegend) and anti-FcεR1α-PE (clone MAR-1, Biolegend). Cells were then washed twice and resuspended in FACS buffer and analyzed with an LSR II flow cytometer (Becton Dickinson). Data were analyzed using FlowJo software (TreeStar, Inc.). Gates were set on singlets and then on live cells. Subsequent gates were based on fluorescence minus one and unstained controls.

### RNA extraction, reverse transcription-PCR (RT-PCR), and real-time PCR

Animal tissues were frozen in liquid nitrogen at necropsy and stored at −80°C. RNA was extracted from tissue as described previously^42^ using Tri-Reagent (Molecular Research Center) according to the manufacturer’s instructions. RNA was treated with DNAseI (Ambion) to remove genomic DNA contamination. For a quantitative analysis of mRNA levels, 1 μg of total RNA from each sample was reverse transcribed in a 50-μl volume (TaqMan reverse transcription [RT] reagent; Applied Biosystems), and 4 μl of cDNA was used for each real-time reaction. RT-PCR was performed using the primers listed in Table S2, SYBR green (Applied Biosystems) and ViiA 7 Real-Time PCR System (Applied Biosystems). Data was analyzed by using the comparative threshold cycle (C_T_) method (Applied Biosystems). Target gene transcription of each sample was normalized to the respective levels of beta-Actin mRNA and represented as fold change over gene expression in control animals.

### Microbiota Sequencing

DNA was extracted from homogenized stool samples using the protocols and reagents specified in the PowerFecal™ DNA Isolation Kit (MoBio Laboratories, Inc.). To facilitate efficient assemblies and longer accurate reads, paired end (PE) libraries were constructed. Bacterial DNA was amplified by PCR enrichment of 16S rRNA encoding sequences from each sample using primers 515F and 806R that flank the V3-V4 hypervariable region and were modified by adding a unique set of 8 oligonucleotide barcodes for purposes of multiplexing.

The resulting PE 16S rRNA amplicons were purified and quantified on an Invitrogen Qubit system. Libraries were normalized and quality assessed on an Agilent Bioanalyzer prior to sequencing with an Illumina MiSeq system. As quality control, sequences containing uncalled bases, incorrect primer sequence, or runs of ≥12 identical nucleotides were removed from the data.

Phylogenetic analysis of the 16S rRNA sequences was accomplished using customized Linux-based command scripts for trimming, demultiplexing, and quality filtering the raw PE sequence data. Using the QIIME^43^ open source software package, the demultiplexed sequences were aligned, clustered, and operational taxonomic units (OTUs) were determined utilizing the Greengenes reference collection (greengenes.lbl.gov). Principal Component Analysis was performed using METAGENassist^44^. Alpha and beta diversity were evaluated using QIIME and the Megan^45^ open source software package. Student’s T-tests were used to identify taxa that displayed statistically significant differences between experimental groups and controls. 16S rRNA sequences are deposited in the Sequence Read Archive (Bioproject PRJNA287262) at the National Center of Biotechnology Information (NCBI).

### Microbiota reconstitution of Germ-free Mice

Control or parasite-infected C57BL6/J mice at 10 days post *P. yoelii* inoculation were euthanized and ceca removed aseptically with cuts 2 cm above and below the cecum to minimize oxygen exposure. Ceca were then transferred to an anaerobic chamber (Bactron I Anerobic Chamber; Sheldon Manufacturing, Cornelius) for processing. The cecal contents from each donor mouse were collected and suspended in 2 ml pre-reduced PBS. Each recipient germ-free Swiss Webster mouse was orally inoculated with 0.2 ml of cecal suspension from one donor mouse and housed in an individual cage for 6 days to allow for microbiota reconstitution.

### Statistical analysis

The statistical significance of differences between groups was determined by a Student’s *t* test on data transformed to a logarithmic scale. A *P* value of 0.05 or less was considered to be significant. All data were analyzed using two-tailed tests.

## Additional Information

**How to cite this article**: Mooney, J. P. *et al.* Inflammation-associated alterations to the intestinal microbiota reduce colonization resistance against non-typhoidal *Salmonella* during concurrent malaria parasite infection. *Sci. Rep.* **5**, 14603; doi: 10.1038/srep14603 (2015).

## Supplementary Material

Supplementary Information

## Figures and Tables

**Table 1:** OTU with significant changes after malaria parasite infection.

Level	Classification	% Abundance duringMalaria			P-value	
		Day 0	Day10	Day15	D0 vsD10	D0 vsD15
INCREASED AT DAY 10						
Phylum	Bacteroidetes	27%	50%	36%	0.004	0.183
Class	Bacteroidia	27%	50%	36%	0.004	0.183
Order	Bacteroidales	27%	50%	36%	0.004	0.183
Order	Turicibacterales	6%	17%	16%	0.001	0.028
Order	RF39	0.4%	1.4%	1.2%	0.025	0.073
Family	Rikenellaceae	14%	30%	20%	0.009	0.036
Family	Turicibacteraceae	6%	17%	16%	0.001	0.755
Genus	Unclassified Rikenellaceae	14%	30%	20%	0.009	0.210
Genus	*Turicibacter*	6%	17%	16%	0.001	0.028
Genus	Unclassified Ruminococcaceae 1	1.5%	3.1%	4.4%	0.007	0.212
Genus	Unclassified Bacteroidales 1	0.0%	0.1%	0.0%	0.024	ns
REDUCED AT DAY 10						
Phylum	Firmicutes	65%	48%	60%	0.001	0.385
Order	Lactobacillales	11%	5%	5%	0.036	0.031
Family	Lachnospiraceae	22%	6%	16%	0.052	0.016
Genus	*Ruminococcus*	1.0%	0.2%	0.4%	0.041	0.093

Table corresponds to data Fig. 3 and S2. All mice were housed in groups.

**Table 2:** Calculation of Implantation Dose.

*S.* Typhimurium					
Dose	Number of miceuninfected	Number ofmice infected	Total		Percentinfected‡
			Uninfected*	Infected†	
30	8	0	24	0	0
320	8	0	16	0	0
3300	6	2	8	2	20%
35000	2	5	2	7	78%
ID_50_ is 10,858 CFU.					

*P. yoelii* + *S*. Typhimurium					
30	5	3	11	3	21%
320	5	3	6	6	50%
3300	1	7	1	13	93%
ID_50_ is 320 CFU.					

C57BL/6 mice were challenged ig 10 days after mock or *Py* infection. Fecal CFU was determined 24 h post challenge. All mice were housed in groups.

^*^The sum of all of the uninfected mice at that dose and higher.

^†^The sum of all of the infected mice at that dose and lower.

^‡^The total infected divided by the sum of the total uninfected and total infected mice multiplied by 100.
