# Supplementary material for: Inflammation-associated alterations to the intestinal microbiota reduce colonization resistance against non-typhoidal Salmonella during concurrent malaria parasite infection
Source: Sci Rep. 2015 Oct 5;5:14603. doi: 10.1038/srep14603 (PMC4592952; doi:10.1038/srep14603)
Supplement: Supplementary Information [file srep14603-s1.pdf]

SUPPLEMENTARY MATERIALS:

Inflammation-associated alterations to the intestinal microbiota reduce colonization resistance against non-typhoidal *Salmonella* during concurrent malaria parasite infection

Jason P. Mooney[1], Kristen L. Lokken[1], Mariana X. Byndloss[1], Michael D. George[1], Eric M. Velazquez[1], Franziska Faber[1], Brian P. Butler[1,2], Gregory T. Walker[1], Mohamed M. Ali[3,4,5], Rashaun Potts[1], Caitlin Tiffany[1], Brian M.M. Ahmer[3,4,6] , Shirley Luckhart[1] and Renée M. Tsolis[1*]

[1]Department of Medical Microbiology & Immunology, University of California at Davis, One Shields Avenue, Davis CA 95616

[2]School of Veterinary Medicine, St. George's University, Grenada, West Indies.

[3] Department of Microbiology, The Ohio State University, Columbus, OH 43210 USA.

[4]Center for Microbial Interface Biology, The Ohio State University, Columbus, OH 43210 USA.

[5]Department of Medical Microbiology and Immunology, Faculty of Medicine, Mansoura University, Mansoura, Egypt

[6]Department of Microbial Infection and Immunity, The Ohio State University, 460 W. 12th Ave, Columbus, OH 43210

*Corresponding author: Department of Medical Microbiology and Immunology, One Shields Avenue, Davis, CA, 95616 Phone 530-854-8497, Fax 530-754-7240

TABLE S1: Histopathology Scoring Criteria.

Score	Edema	Epithelial damage	Infiltration by mononuclear cells
0	Absent	Absent	Absent
1	Mild focal accumulation of fluids in the lamina propria (edema)	Mild focal loss of goblet cells and hyperplasia of undifferentiated enterocytes	Mild focal infiltration of macrophages/dendritic cells and/or lymphocytes in the lamina propria (5-10 cells/high power field)
2	Moderate focal to multifocal accumulation of fluids in the lamina propria (edema)	Moderate focal to multifocal loss of goblet cells and hyperplasia of undifferentiated enterocytes	Moderate focal to multifocal infiltration of macrophages/dendritic cells and/or lymphocytes in the lamina propria (10-20 cells/high power field)
3	Severe multifocal to severe accumulation of fluids in the lamina propria (edema)	Severe multifocal to diffuse loss of goblet cells and hyperplasia of undifferentiated enterocytes	Severe multifocal to diffuse infiltration of macrophages/dendritic cells and/or lymphocytes in the lamina propria (20-40 cells/high power field)

TABLE S2: Quantitative real-time PCR primers used in this study.

Gene	Sequences (5'→3')
β-actin	CCAGGGAGGAAGAGGATGCGG
	GCTGAGAGGGAAATCGTGCGTG
Il10	GGTTGCCAAGCCTTATCGGA
	ACCTGCTCCACTGCCTTGCT
Ifnγ	CAACAGCAAGGCGAAAAAGGATGC
	CCCCGAATCAGCAGCGACTCC
S100a8	TGTCCTCAGTTTGTGCAGAATATAAA
	TCACCATCGCAAGGAACTCC
S100a9	GGTGGAAGCACAGTTGGCA
	GTGTCCAGGTCCTCCATGATG
Cxcl1	GCTTGCCTTGACCCTGAAGCTC
	TGTTGTCAGAAGCCAGCGTTCAC

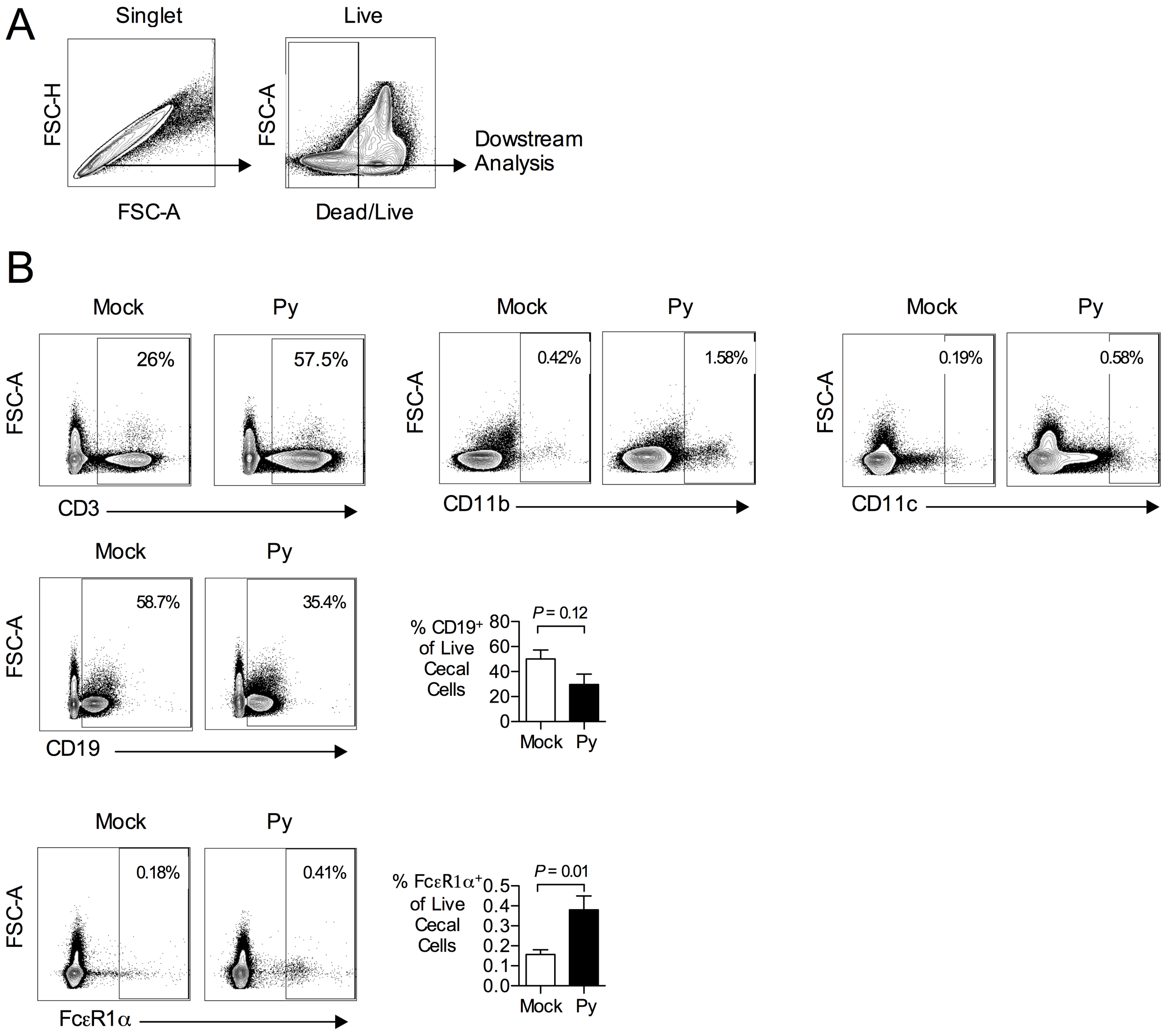

Figure S1: Cellular infiltration into the intestinal mucosa during *P. yoelii* infection. Flow cytometry analysis of cell suspensions prepared from the cecum of mice at 10d after inoculation with *P. yoelii*. **(A)** Gating scheme used in this study: singlet forward side-scatter (FSC)-height (H) and area (A) followed by dead/live discrimination. **(B)** Representative dot plots of CD3, CD11b and CD11c expressing cells shown in Figure 1D. In addition, the proportion of B cells (CD19) and IgE receptor expressing cells (FcεR1α) was determined. Data shown as mean+SEM of n=5. Significance of differences between experimental groups was determined by a Student's *t* test on logarithmically transformed data.

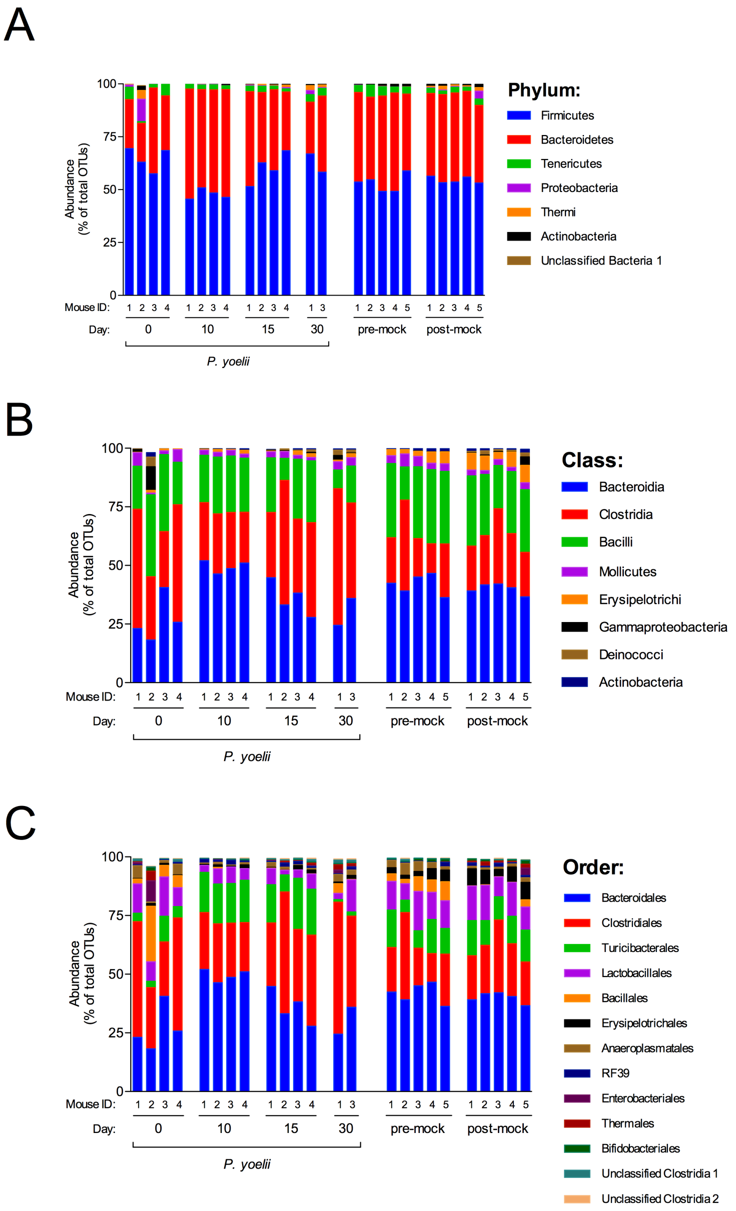

Figure S2: Changes in microbial communities after *P. yoelii* infection. Microbial community analysis was performed by Illumina MiSeq sequencing of 16S rRNA from fecal pellets of C57BL/6 mice before and after malaria parasite infection. Parallel analysis was performed on control mice (received in the same shipment as experimental mice), sampled two weeks apart. Average abundance of microbial communities was determined by percent OTU readings at days 0, 10, 15 (n=4) and 30 (n=2), and displayed as percent abundance within the levels of **(A)** Phylum, **(B)** Class, and **(C)** Order. See Table 1 for significant changes.

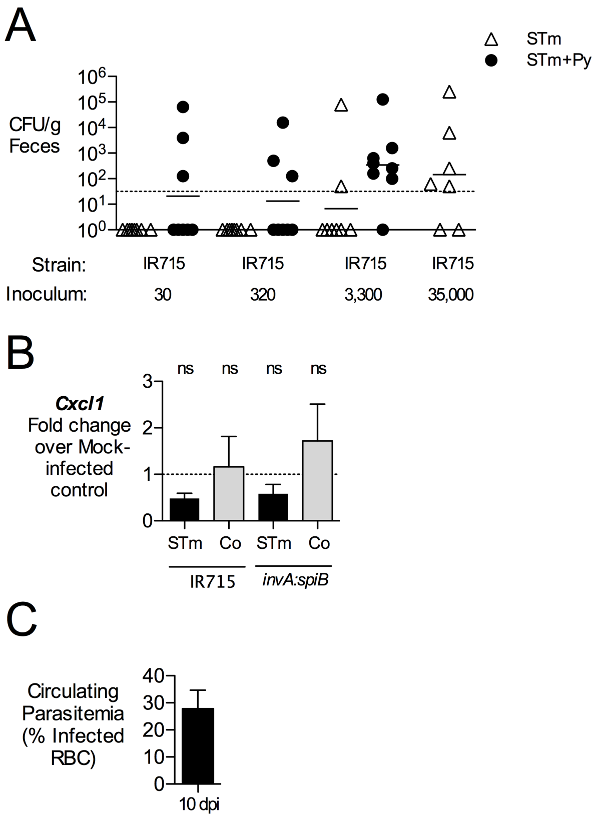

Figure S3: Reduction of colonization resistance against *S.* Typhimurium in *P. yoelii*-infected mice. (A) Colonization levels of *S.* Typhimurium strain IR715 in feces of control (STm) or *P. yoelii*-infected mice (STm + Py) that were used to calculate implantation dose in Table 3. Significance of differences between groups was determined using a Student's *t* test. **(B)** Abundance of transcripts encoding neutrophil chemoattractant *Cxcl1* in cecal tissue from control (STm) mice or *P. yoelii*-infected mice (Co) shown in Fig 4B, showing lack of this transcript at 1d after *S.* Typhimurium infection. Data shown as fold change over mock-treated mice with mean+SEM (n=5). **(C)** Parasitemia of donor mice for fecal microbiota transplant was monitored on Giemsa-stained blood smears and the percentage of iRBC is displayed as mean+SEM (n=3). NS indicates no significance (P>0.05) when compared to mock-treated mice as determined by Student's *t* test on logarithmically transformed data.
